# Supplementary material for: Robo signalling controls pancreatic progenitor identity by regulating Tead transcription factors
Source: Nat Commun. 2018 Nov 30;9:5082. doi: 10.1038/s41467-018-07474-6 (PMC6269453; doi:10.1038/s41467-018-07474-6)
Supplement: Supplementary file 1 — Supplementary Information [file 41467_2018_7474_MOESM1_ESM.pdf]

SUPPLEMENTARY INFORMATION

Robo signalling controls pancreatic progenitor identity by regulating Tead transcription factors

Sophie Escot[1], David Willnow[1,2], Heike Naumann[1], Silvia Di Francescantonio[1], Francesca M. Spagnoli[1,2,3*]

[1]Lab. of Molecular and Cellular Basis of Embryonic Development, Max-Delbrueck Center for Molecular Medicine, Robert-Roessle Strasse 10, Berlin 13125, Germany; [2] Berlin Institute of Health (BIH), Berlin, Germany; [3]Centre for Stem Cell and Regenerative Medicine, King's College London, Great Maze Pond, London SE1 9RT, United Kingdom.

*corresponding author: francesca.spagnoli@kcl.ac.uk

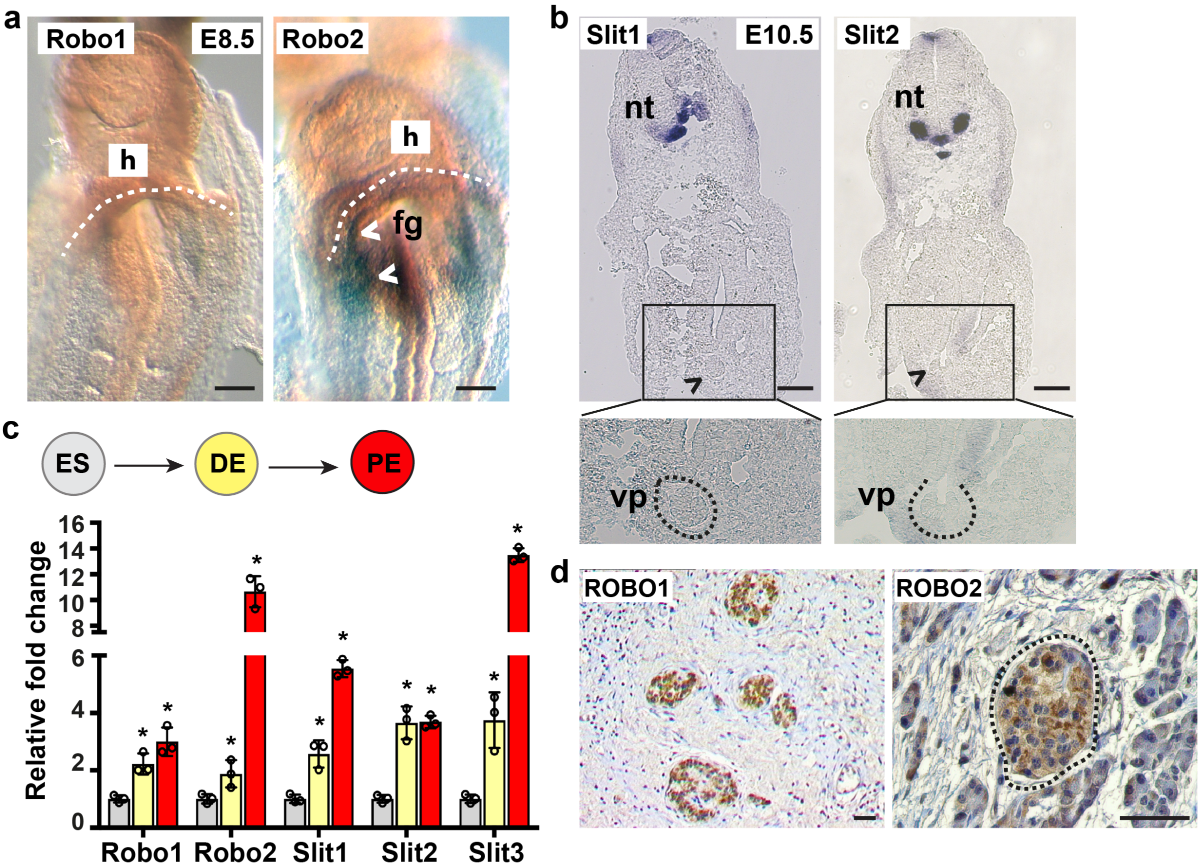

Supplementary Figure 1. Robo genes are expressed in pancreatic cells throughout development until adult age. (**a**) X-gal-stained E8.5 embryos expressing β-galactosidase (β-gal) from the Robo1 and Robo2 loci in the respective mutant alleles[1, 2]. Dotted lines demarcate ventral foregut (fg) epithelium; arrowheads indicate expression in the ventral foregut. h, heart. Scale bars, 100µm. (**b**) *In situ* hybridisation analysis of *Slit1* and *Slit2* on E10.5 mouse cryosections. In line with previous reports[2, 3], Slit1 and Slit2 are abundantly expressed in the neural tube (nt) at this embryonic stage, but absent in the pancreatic epithelium or mesenchyme (arrowheads). Inset below show higher magnification of boxed area; dashed lines demarcate the ventral pancreatic bud (vp). Scale bar, 100 µm. (**c**) Schematics of pancreatic differentiation of mESC. RT-qPCR analysis evaluating *Robo1* and *Robo*2 as well as *Slits* gene expression at definitive endoderm (DE) and pancreatic endoderm (PE) stages of differentiation. Untreated mESC cells were used as control (ES). Data were normalized to that of SDHA and represented as relative fold change. Values shown are mean ± s.e.m. (n=3). *P<0.05, two-tailed unpaired t-test. Source data are provided as a Source Data file. (**d**) Representative immunohistochemistry images of ROBO1 and ROBO2 in human pancreatic tissue. Both ROBO1 and ROBO2 were abundant in human pancreatic islets, which is consistent with previous reports in the adult mouse[4, 5]. Scale bars, 100 µm.

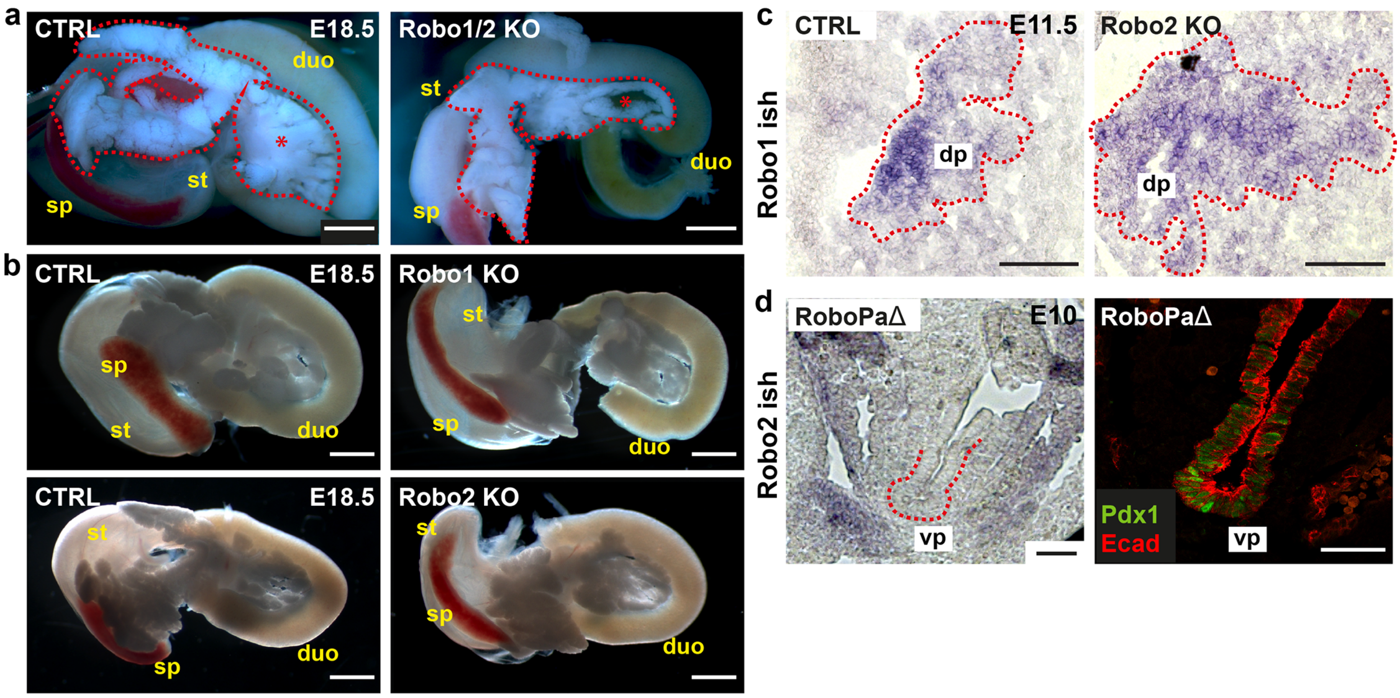

Supplementary Figure 2. Pancreas size and gastrointestinal tract in *Robo1* and *Robo2* single mutant embryos. (**a**) Pancreas gross morphology at E18.5 of CTRL and Robo1/2 KO. The head of the pancreas is connected to the duodenum, while the tail is next to the stomach (asterisk). Red dashed lines delineate the whole pancreas. (**b**) Pancreas gross morphology at E18.5 of CTRL and Robo1 KO and Robo2 KO embryos. duo, duodenum; sp, spleen; st, stomach. Scale bar, 1 mm. (**c**) In situ hybridisation analysis of *Robo1* on E11.5 mouse cryosections of CTRL and Robo2 KO pancreas. Red dashed lines delineate the pancreatic epithelium. Scale bar, 100 µm. (**d**) *In situ* hybridisation analysis of *Robo2* on E10 mouse cryosections of Robo$^{Pa\Delta}$ pancreas (Left). Red dashed lines delineate the ventral pancreatic epithelium (vp), which is depleted of Robo2 upon Cre recombination. Scale bars, 100 µm. Right, consecutive sections stained using antibodies against Pdx1 and E-cadherin. Scale bars, 50 µm.

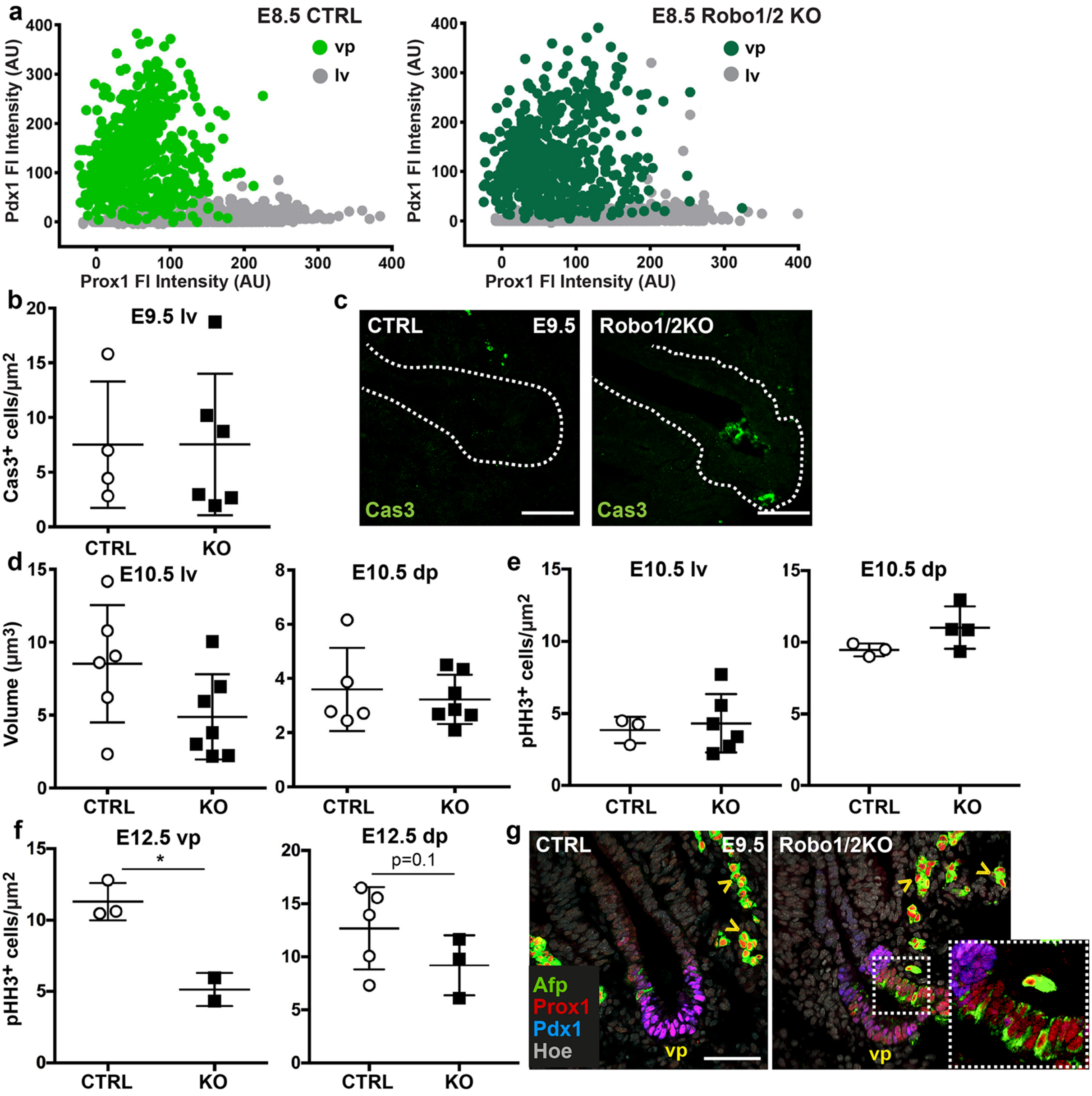

Supplementary Figure 3. Robo signalling specifically affects ventral pancreatic progenitors at early stages. (**a**) Single cell measurement of fluorescence intensity of Prox1 and Pdx1 in hepatic endoderm (grey circle) and prospective ventral pancreatic endoderm of E8.5 CTRL (light green circle) and Robo1/2 KO (dark green circle) embryos. Fluorescence intensity was measured with Fiji and values normalized within each embryo. FI, fluorescence intensity; AU, arbitrary units. n=3 per each genotype. (**b**) Quantification of Cas3$^+$ cells in liver bud of E9.5 CTRL (n=4) and Robo 1/2 KO (n=6) embryos. Number of Cas3$^+$ cells was normalised to sum of epithelial areas. (**c**) Single channels of Cleaved-Caspase3 (Cas3) IF images of E9.5 CTRL and Robo1/2 KO cryosections shown in Fig. 3a. Scale bars, 50 µm. (**d**) Quantification of E10.5 liver (c) and dorsal pancreas (dp) volumes from confocal images of CTRL (n=6) and Robo1/2 KO (n=7) embryos. Error bars represent ± s.e.m. (**e**) Quantification of proliferation in liver and dorsal pancreatic buds of E10.5 CTRL and Robo1/2 KO cryosections. (**f**) Quantification of proliferation in ventral and dorsal pancreatic epithelium of E12.5 CTRL and Robo1/2 KO cryosections. Number of

pHH3$^+$ cells was normalised to the sum of the Ecad+ epithelium area (μm^2). Error bars represent ± s.e.m. Two tailed Student's t test *$P<0.05$. (**g**) Representative IF images of E9.5 CTRL and Robo1/2 KO cryosections stained for Alpha-fetoprotein (Afp), Pdx1 and Prox1. Hoechst (Hoe) was used as nuclear counterstain. Scale bar, 50 μm.

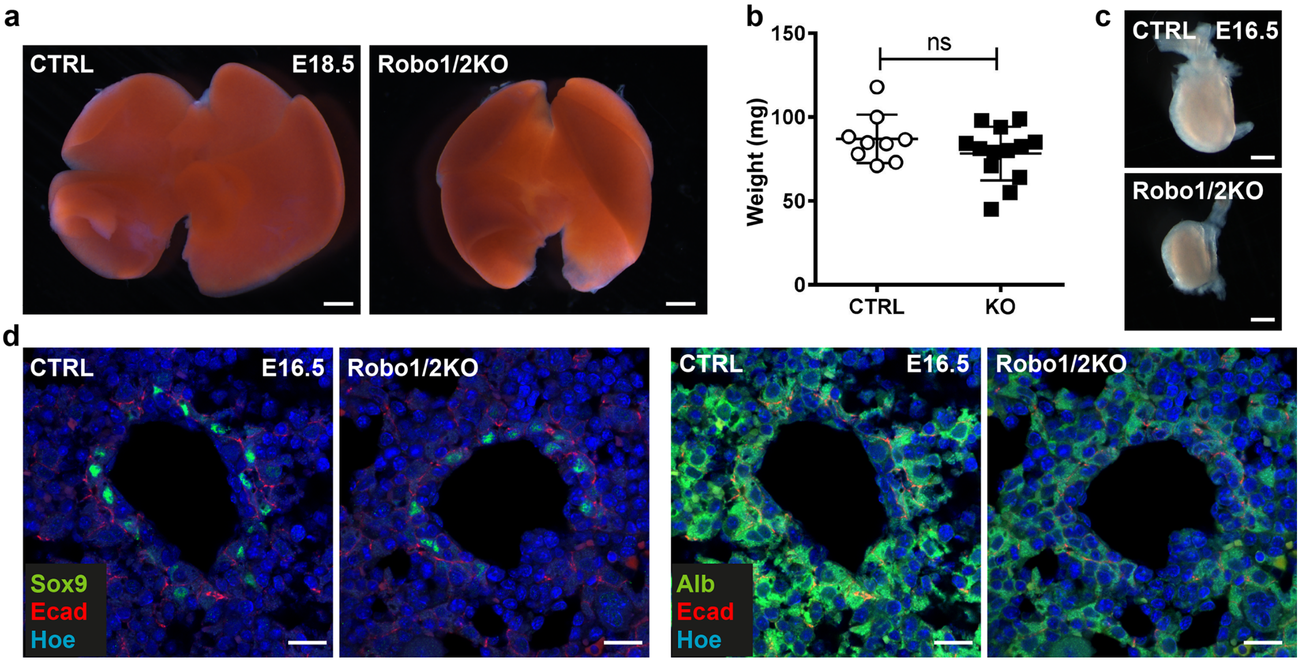

Supplementary Figure 4. Robo signalling in liver and gall bladder development. (**a**) Liver gross morphology control (CTRL) and Robo1/2 KO embryos at E18.5. Scale bars, 1 mm. (**b**) Quantification of liver weight in control (CTRL; n=9) and Robo1/2 KO (KO; n=13) E18.5 mouse embryos. Error bars represent ± s.e.m. (**c**) Gall bladder gross morphology at E16.5. Robo1/2 KO shows reduction of gall bladder size. Scale bars, 1mm. (**d**) Immunofluorescence analysis of Sox9, Albumin (Alb), E-cadherin (Ecad) on CTRL and Robo1/2 KO E18.5 mouse liver cryosections. Hoechst (Hoe) used as nuclear counterstain. *Robo1* and *Robo2* ablation does not affect liver lineage differentiation. Scale bars, 20 μm.

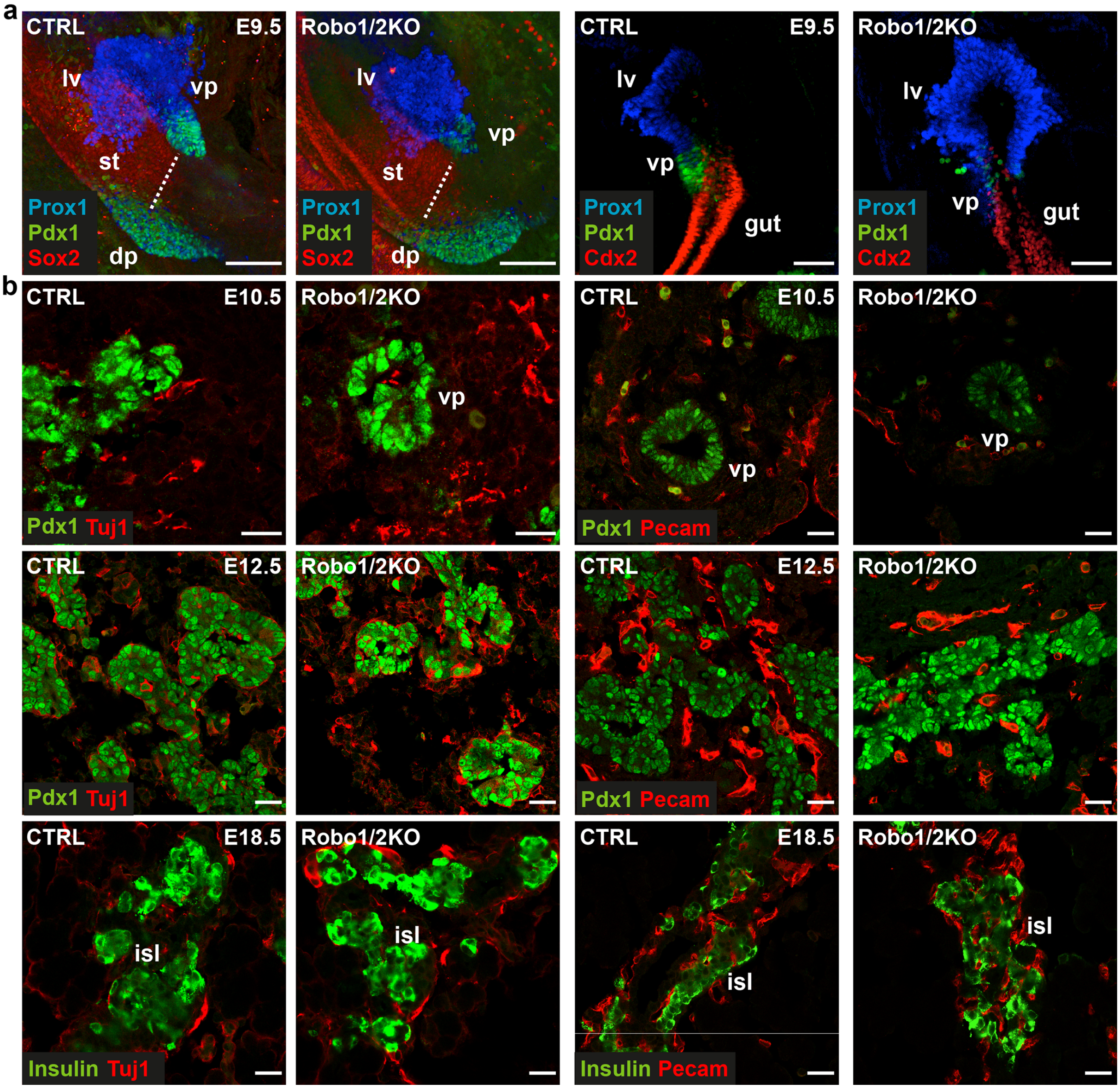

Supplementary Figure 5. Ablation of *Robo1* and *Robo2* does not affect pancreas innervation and vascularisation. (**a**) Representative confocal maximum intensity z-projections of whole-mount IF for Prox1, Pdx1 along with Sox2 (left panels) or Cdx2 (right panels) on E9.5 CTRL and Robo1/2 KO embryos. Dashed line marks border of prospective stomach (st), positive for Sox2. Anterior and posterior patterning of the primitive gut is not altered in Robo1/2 KO embryos. dp, dorsal pancreatic bud; lv, liver bud; vp, ventral pancreatic bud. Scale bars, 50µm. (**b**) Immunofluorescence analysis with antibodies against Pdx1, insulin, Pecam (endothelial cells) and Tuj1 (neural precursor cells) on E10.5, E12.5 and E18.5 CTRL and Robo1/2 KO pancreas cryosections. Notably, Robo1/2 KO newborn islets (isl) displayed disrupted architecture (see also Fig. 1e), which is in line with recent findings highlighting the role of Robo receptors in endocrine cell clustering[4]. Scale bars, 20 µm.

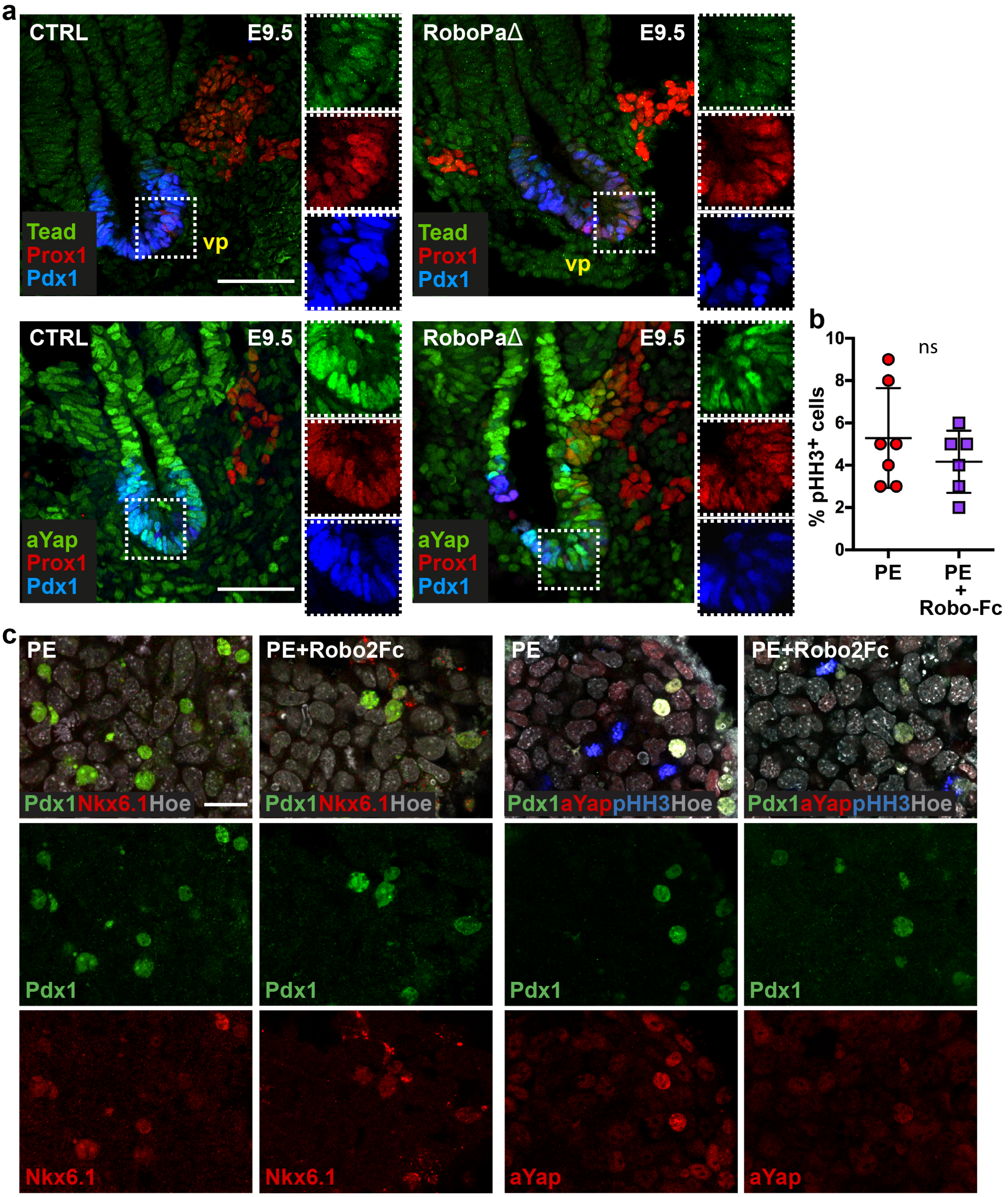

Supplementary Figure 6. Characterisation of Tead and Yap signalling in Robo$^{Pa\Delta}$ pancreas and mESCs. (**a**) Immunofluorescence analysis of Pdx1, Prox1, Tead (top panel) or active-Yap (bottom panel) on E9.5 CTRL and Robo$^{Pa\Delta}$ pancreas cryosections. Insets show boxed area at higher magnification and single channels. vp, ventral pancreas. (n=3) Scale bars, 50µm. (**b**) Percentage of dividing cells was calculated by counting pHH3+ cells relative to Hoechst+ cells in untreated PE cultures and PE + Robo2-Fc treated cultures. Note the number of pHH3-positive cells is affected in the treated PE-cultures, even though without reaching a significance level. PE, pancreatic endoderm-derived from mESCs. Error bars represent ± s.e.m. (**c**) Representative IF images of PE and PE + Robo2-Fc stained with Pdx1, Nkx6.1,

a-Yap and pHH3 antibodies. Hoechst (Hoe) was used as nuclear counterstain. Scale bars, 20μm.

Supplementary Table 1. Primary antibody list

Antibody	Host Species	Dilution	Source
anti-alphaFETOPROTEIN	Rabbit	1:500	Dako A000829-2
anti-ALBUMIN	Rabbit	1:500	Dako A0001
anti-AMYLASE	Rabbit	1:500	Sigma A8273
anti-cleaved CASPASE 3	Rabbit	1:300	CST 9661
anti-E-CADHERIN	Rat	1:1000	Sigma U3254
anti-CK19	Mouse	1:400	Abcam ab133496
Anti-F-ACTIN (Phalloidin)	488-Alexa-conj.	1:100	Molecular Probes
anti-GFP	Chicken	1:400	Aves GFP-1020
anti-GLUCAGON	Rabbit	1:500	Immunostar Inc. 20076
anti-INSULIN	Guinea pig	1:250	Invitrogen 18-0067
Anti-LAMININ	Rabbit	1:1000	Sigma L9393
Anti-NKX6.1	Mouse	1:500	Hybridoma Bank
anti-panTEAD	Rabbit	1:100	CST 13295S
anti-PDX1	Guinea pig	1:500	Abcam ab47308
anti-phosphoHISTONE H3	Rabbit	1:200	Millipore 06-570
anti-phosphoHISTONE H3	Mouse	1:100	CST 9706
anti-PROX1	Rabbit	1:200	RELIATech 102-PA32S
anti-ROBO1	Goat	1:200	R&D Systems AF1749
anti-ROBO2	Goat	1:200	R&D Systems AF3147
anti-SOX17	Goat	1:100	R&D Systems AF1924
anti-SOX9	Rabbit	1:500	Millipore AB5535
anti-active YAP1	Rabbit	1:500	Abcam ab205270

Supplementary Table 2. Primer sequences used for quantitative real-time PCR

Gene symbol	Forward primer	Reverse primer
36B4	GGC CCT GCA CTC TCG CTT TC	TGC CAG GAC GCG CTT GT
Axin2	GCTGCGCTTTGATAAGGTCC	AGCCTCCTCTCTTTTACAGCA
Ctgf	CCCTAGCTGCCTACCGACT	CATTCCACAGGTCTTAGAACAG G
Foxa2	CAT CCG ACT GGA GCA GCT A	GCG CCC ACA TAG GAT GAC
Hes1	CAACACGACACCGGACAAAC	GGAATGCCGGGAGCTATCTT
Hey1	TGGTACCCAGTGCCTTTGAGAA	AACTCCGATAGTCCATAGCCAG
HNF1b	GGCCTACGACCGGCAAAAGA	GGGAGACCCCTCGTTGCAAA
Jun	CCAGCAACTTTCCTGACCCA	TTTGCAAAAGTTCGCTCCCG
Nkx6.1	ACTTGGCAGGACCAGAGAG	GCGTGCTTCTTTCTCCACTT
Pdx1	CATCGCGCCACTGCGAC	TTTCTTCTTGTGCGACGCC
Prox1	CCGACATCT CAC CTTATTGAG	TGCGAGGTA ATG CAT CTG TTG
Robo1	GCTGGCGACATGGGATCATA	AATGTGGCGGCTCTTGAACT
Robo2	CGAGCTCCTCCACAGTTTGT	GTAGGTTCTGGCTGCCTTCT
Sdha	TGT TCA GTT CCA CCC CAC A	TCT CCA CGA CAC CCT TCT GT
Sox17	GTA AAG GTG AAA GGC GAG GTG	GTC AAC GCC TTC CAA GAC TTG
Tead2	CTGGACAGGTAGCGAGGAAG	GTTCCGGCCATACATCTTGC

References

1. Grieshammer, U. *et al.* SLIT2-mediated ROBO2 signaling restricts kidney induction to a single site. *Dev Cell* **6**, 709–717 (2004).
2. Long, H. *et al.* Conserved roles for Slit and Robo proteins in midline commissural axon guidance. *Neuron* **42**, 213–223 (2004).
3. Brose, K., Tessier-Lavigne, M. Slit proteins: key regulators of axon guidance, axonal branching, and cell migration. *Curr. Opin. Neurobiol.* **10**, 95–102 (2000).
4. Adams, M.T., Gilbert, J.M., Hinojosa Paiz, J., Bowman, F.M. & Blum, B. Endocrine cell type sorting and mature architecture in the islets of Langerhans require expression of Roundabout receptors in β cells. *Scientific Reports* **8**, 10876 (2018).
5. Yang, Y.H.C., Manning Fox, J.E., Zhang, K.L., MacDonald, P.E. & Johnson, J.D. Intraislet SLIT-ROBO signaling is required for beta-cell survival and potentiates insulin secretion. *Proc. Natl. Acad. Sci. U. S. A.* **110**, 16480–16485 (2013).
