# Supplementary material for: Robo signalling controls pancreatic progenitor identity by regulating Tead transcription factors
Source: Nat Commun. 2018 Nov 30;9:5082. doi: 10.1038/s41467-018-07474-6 (PMC6269453; doi:10.1038/s41467-018-07474-6)
Supplement: Supplementary file 5 — Description of Additional Supplementary Files [file 41467_2018_7474_MOESM5_ESM.docx]

**Title:** Source Data 1

**Description:** Source Data 1 displays the relative quantification (RQ) values of RT-qPCR shown as charts in Fig. 1b, Fig. 6e, Fig. 7a and Supplementary Fig. 1c.
